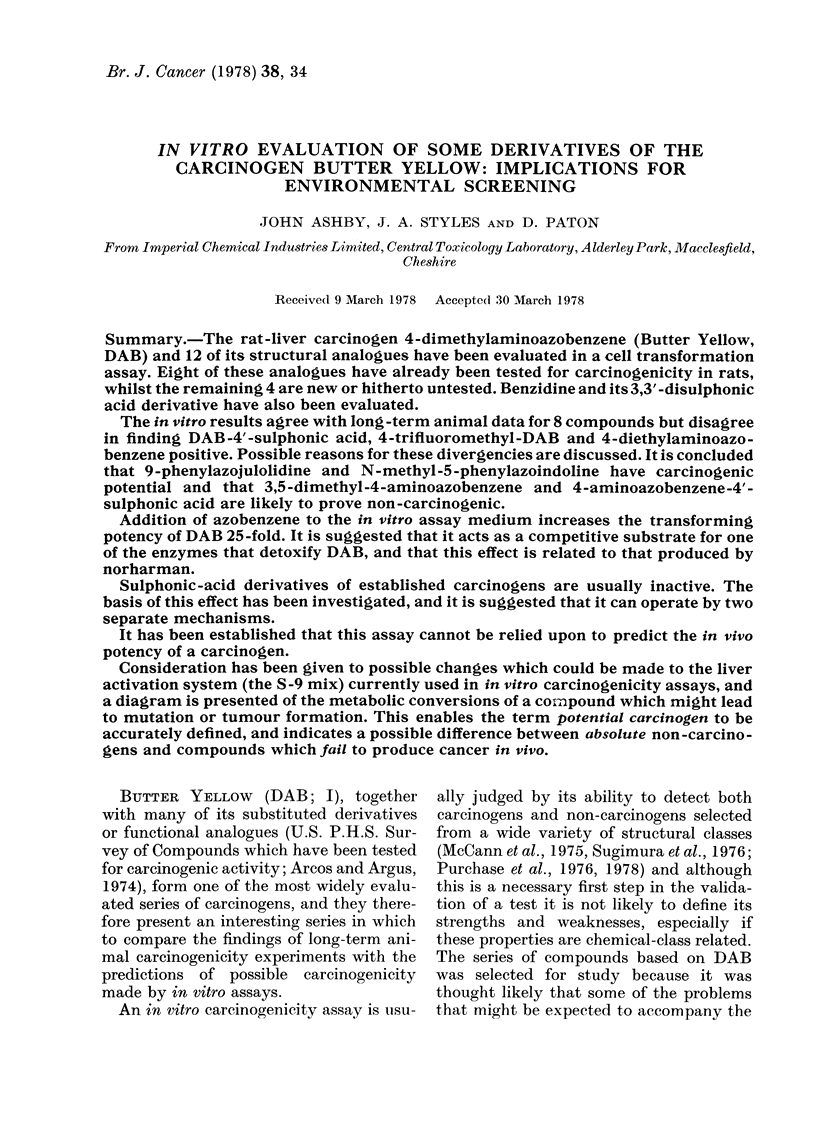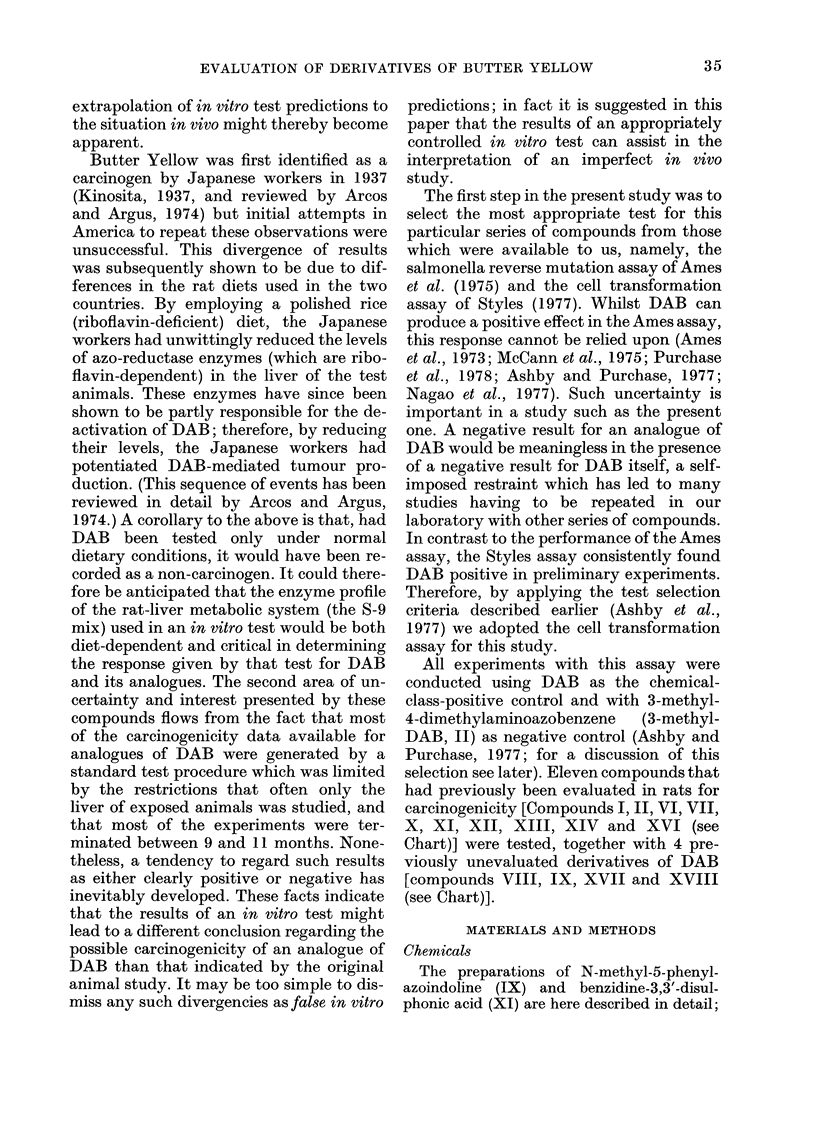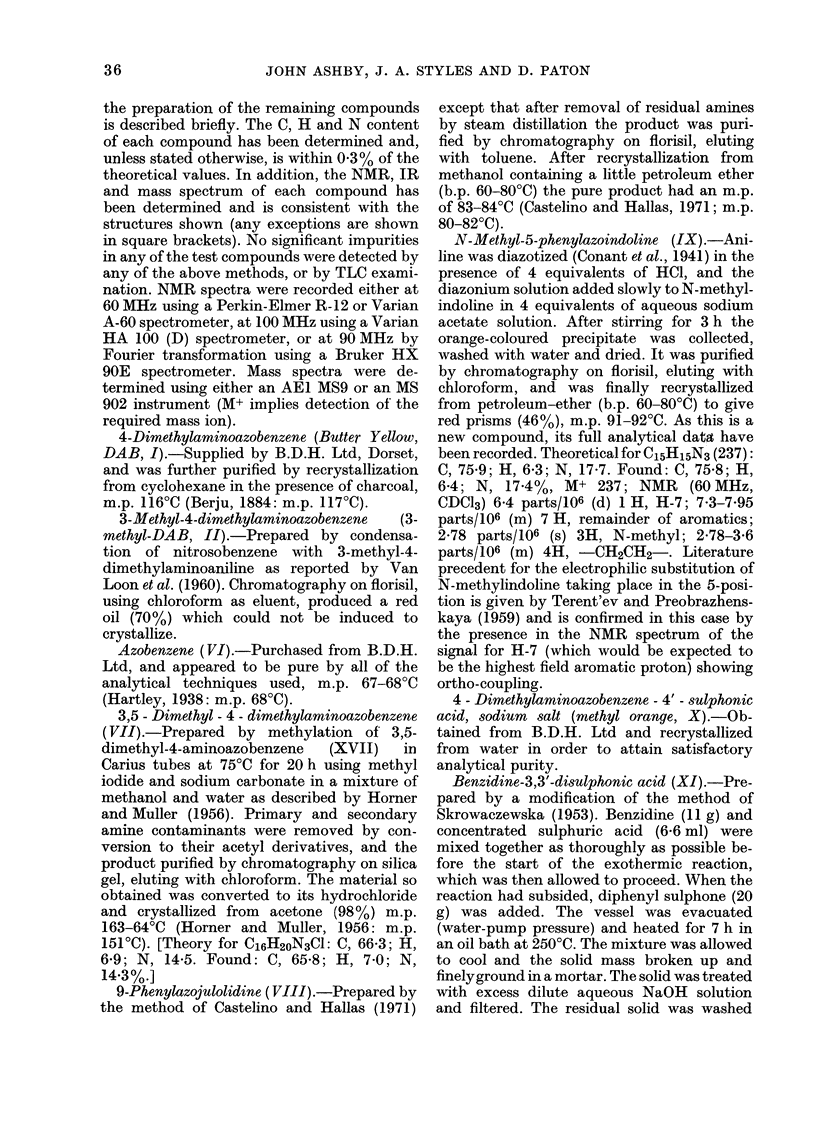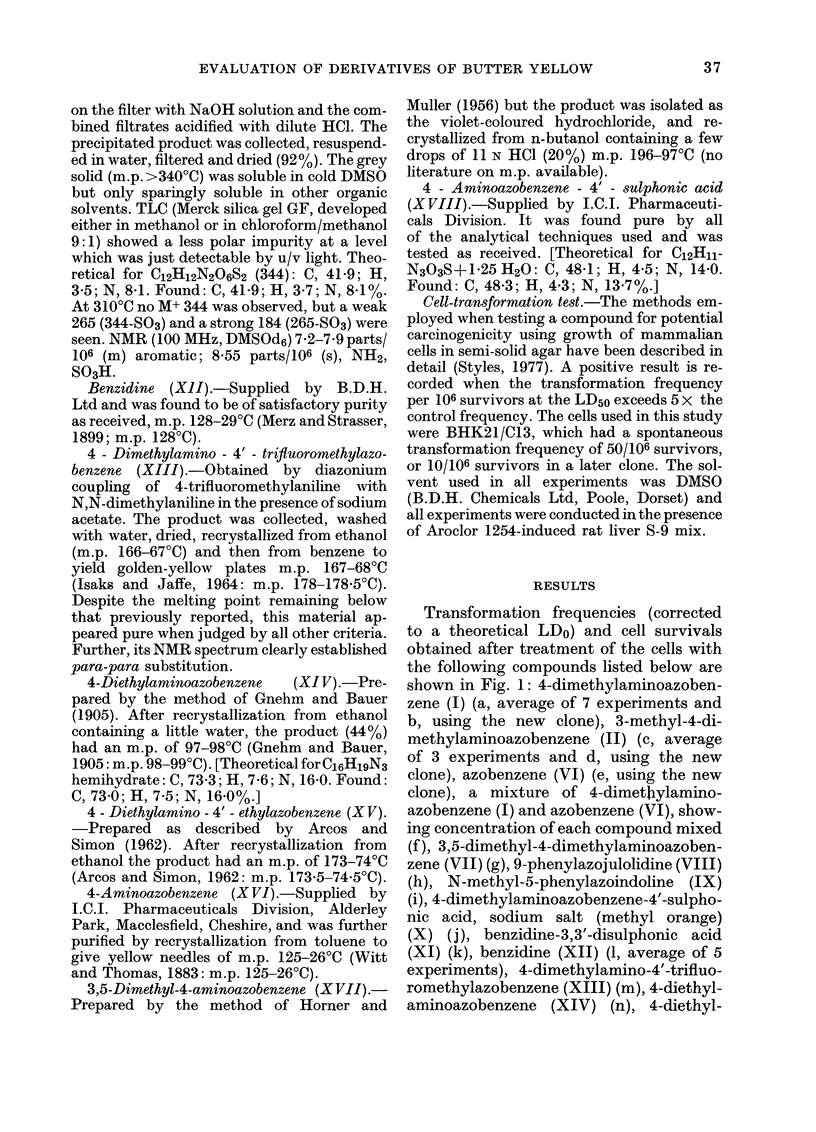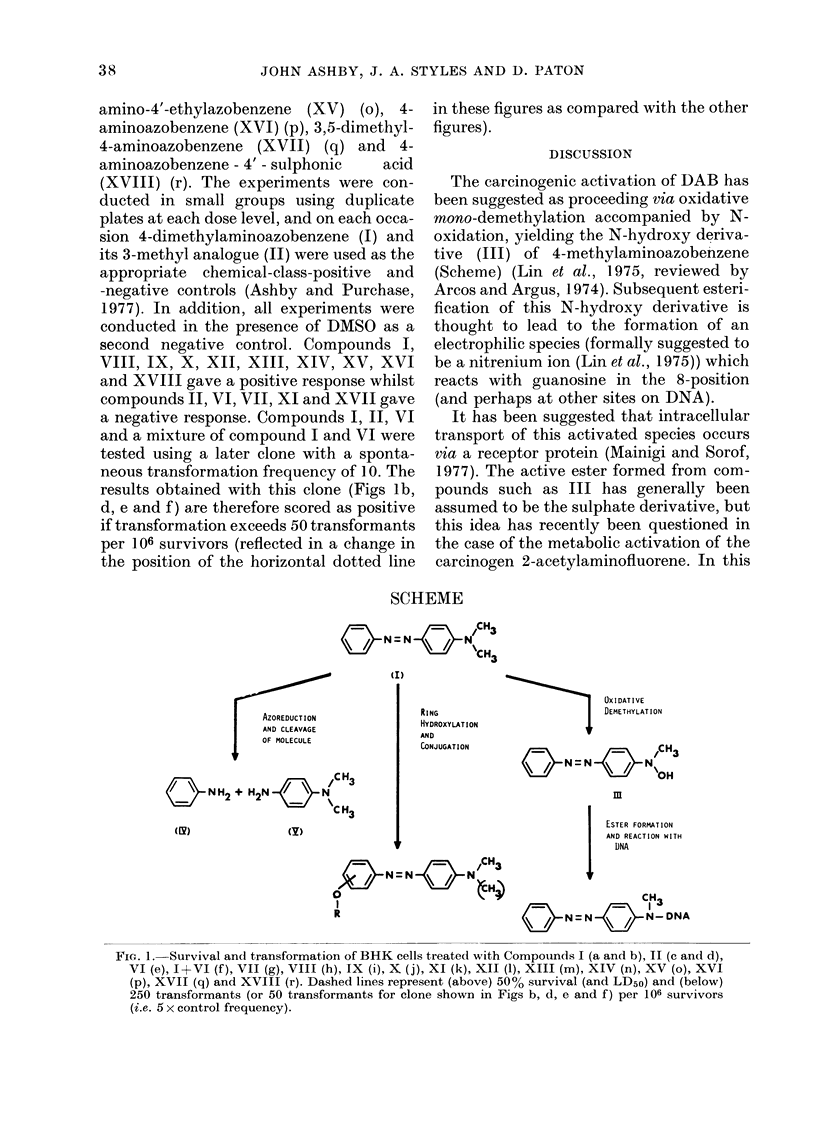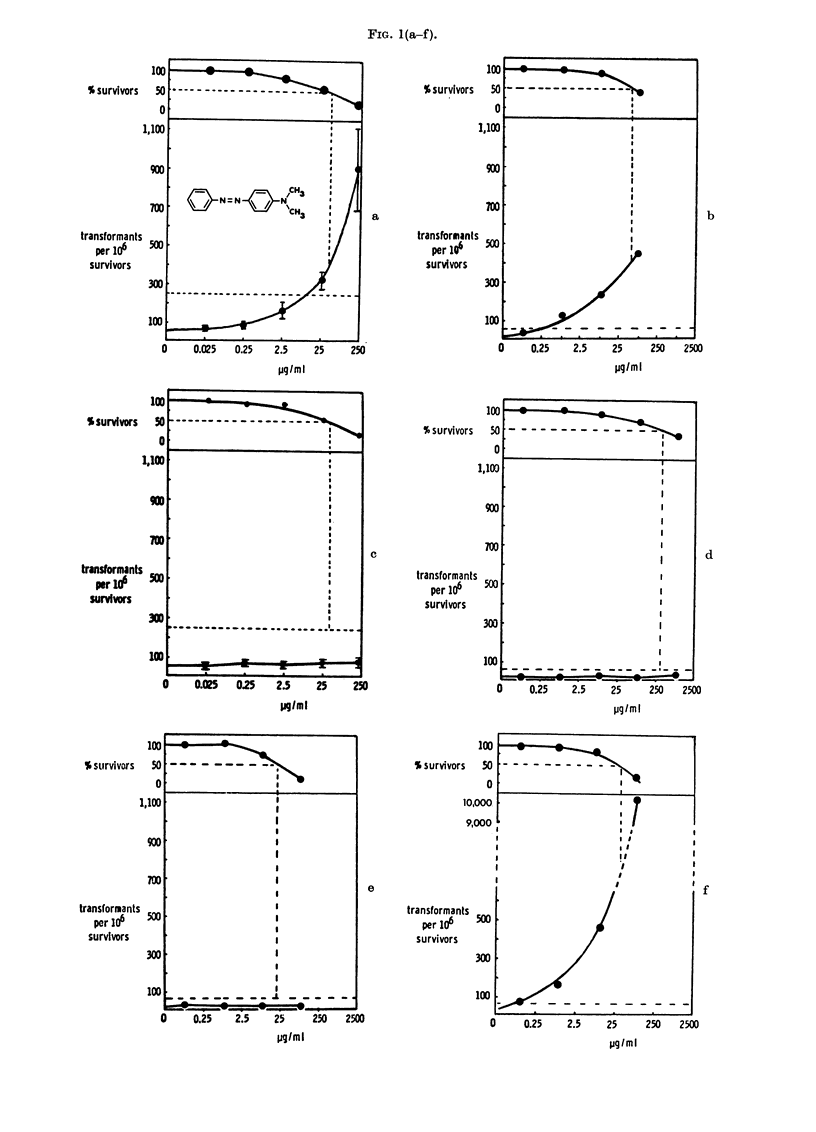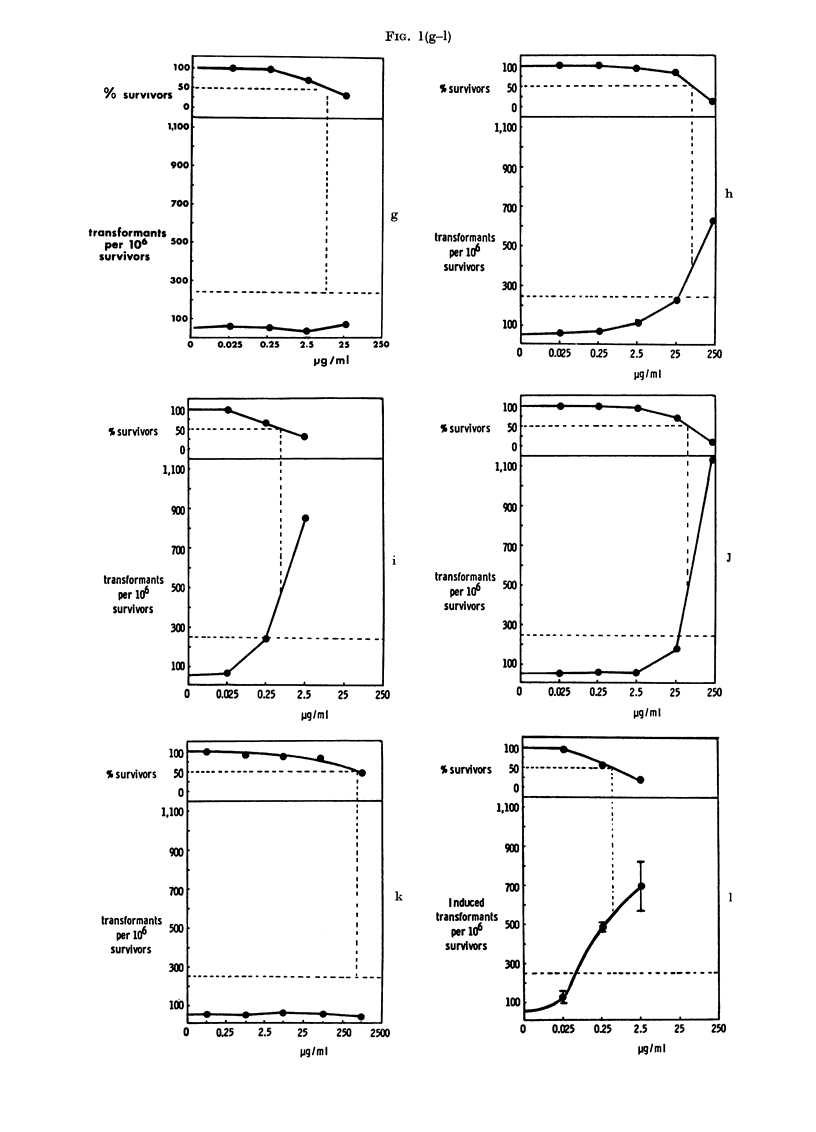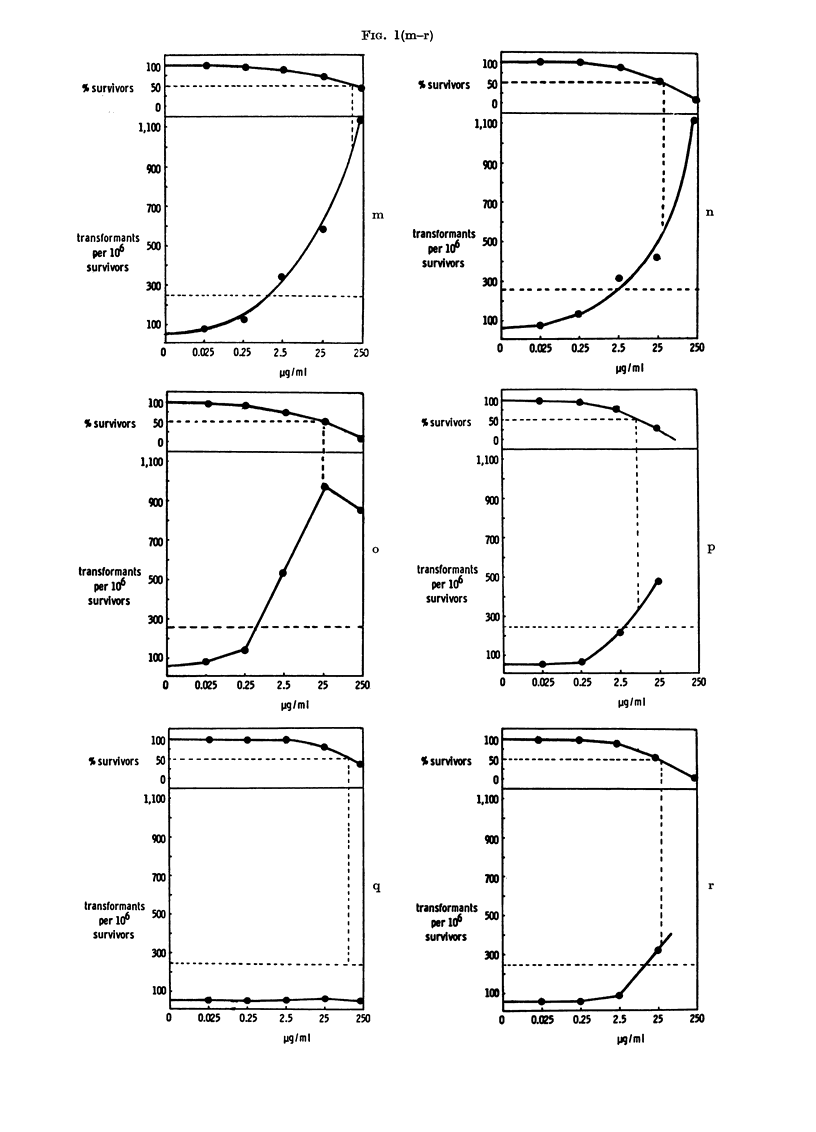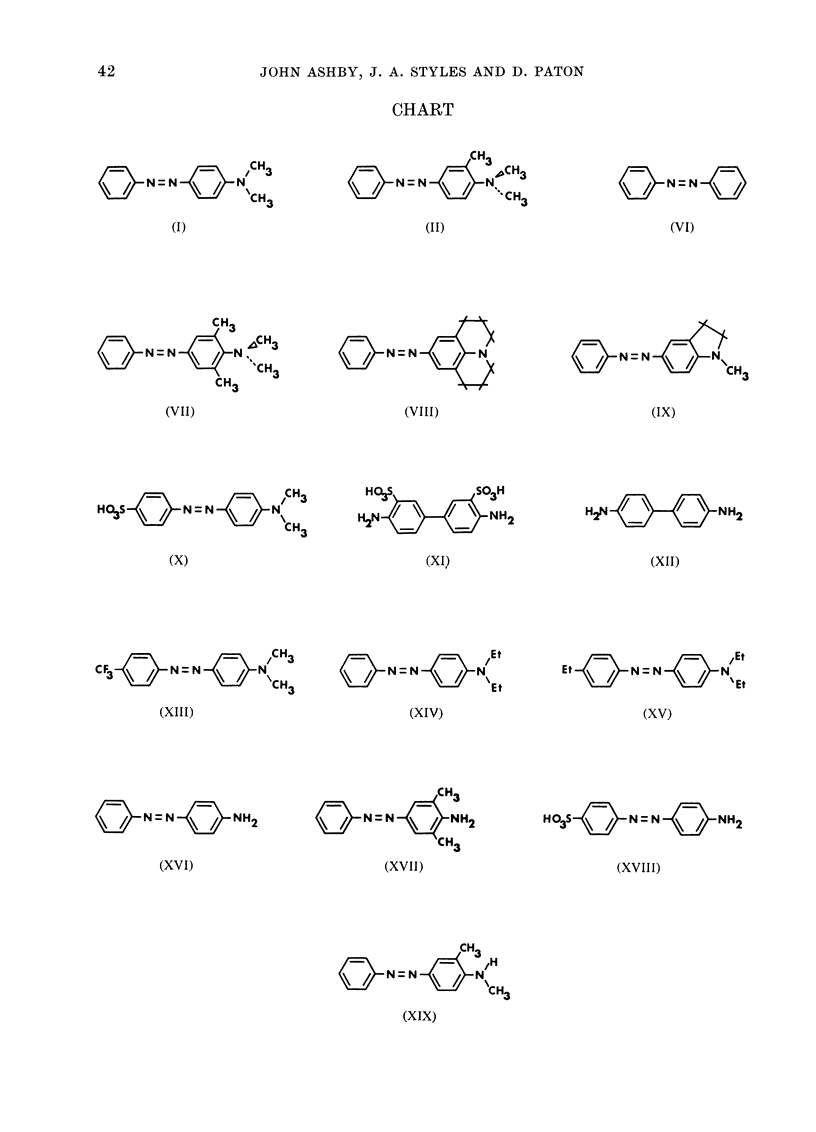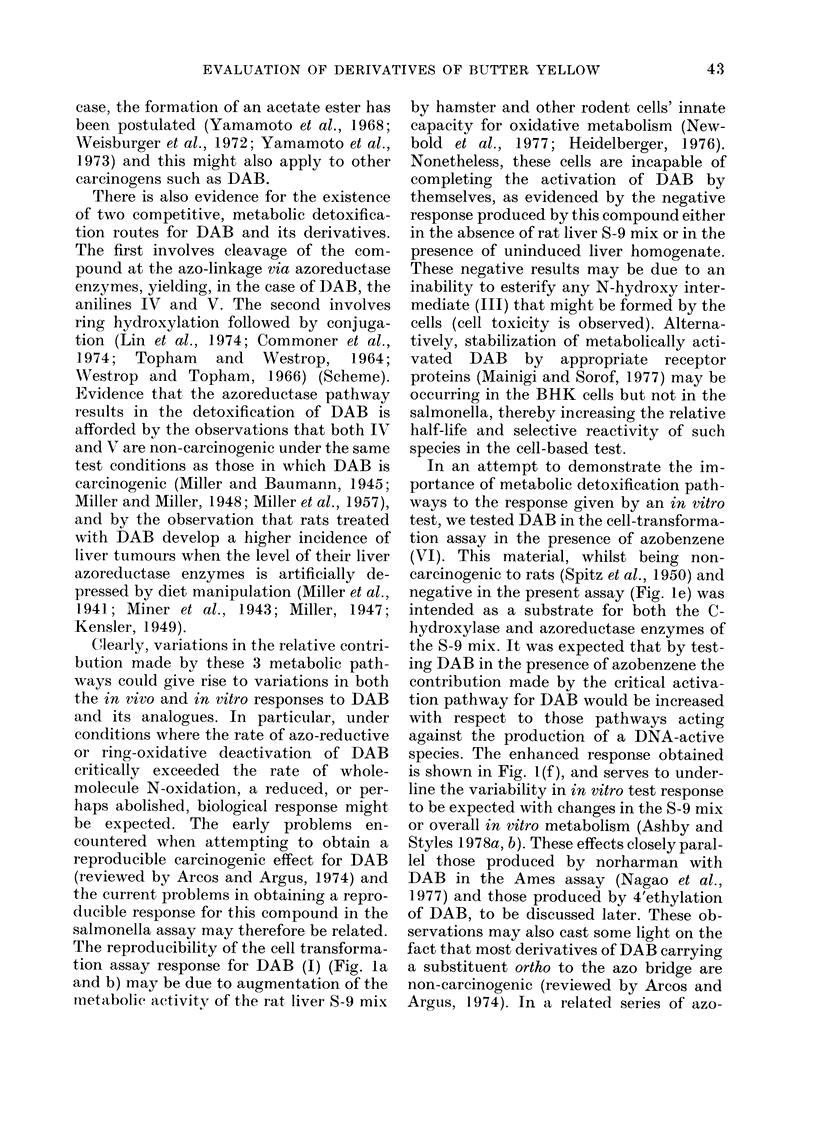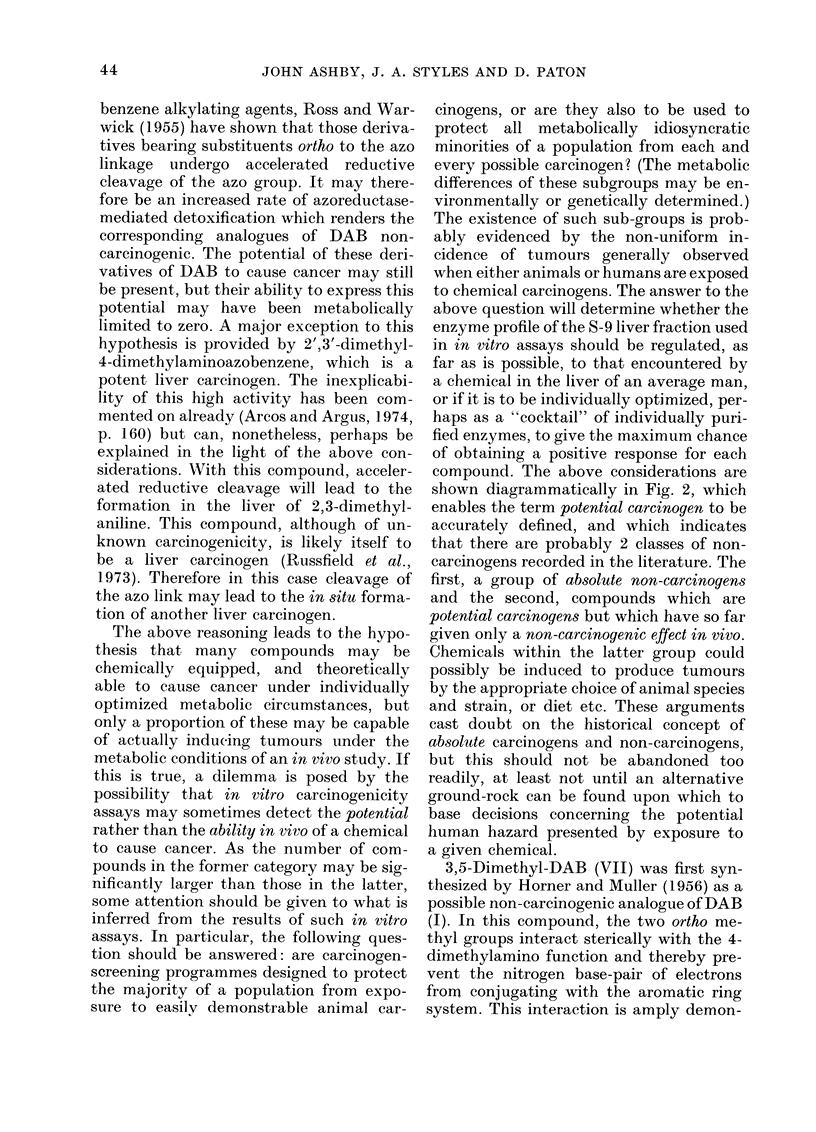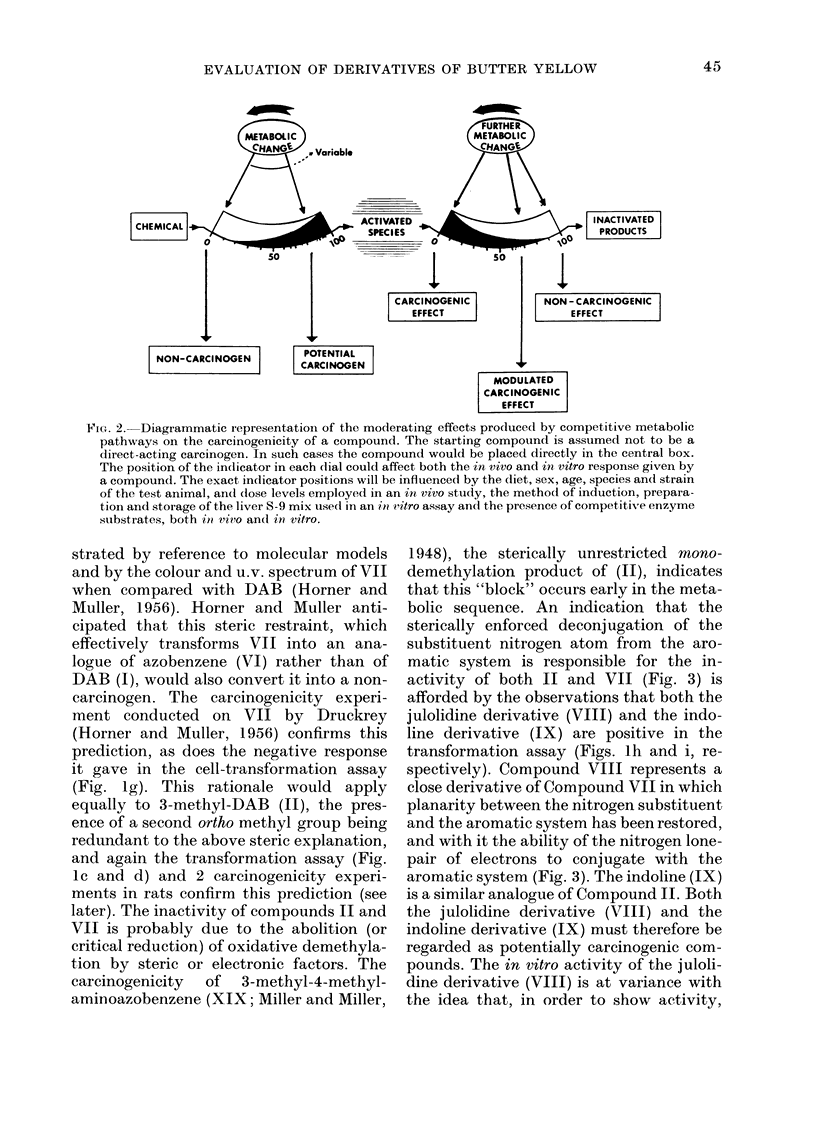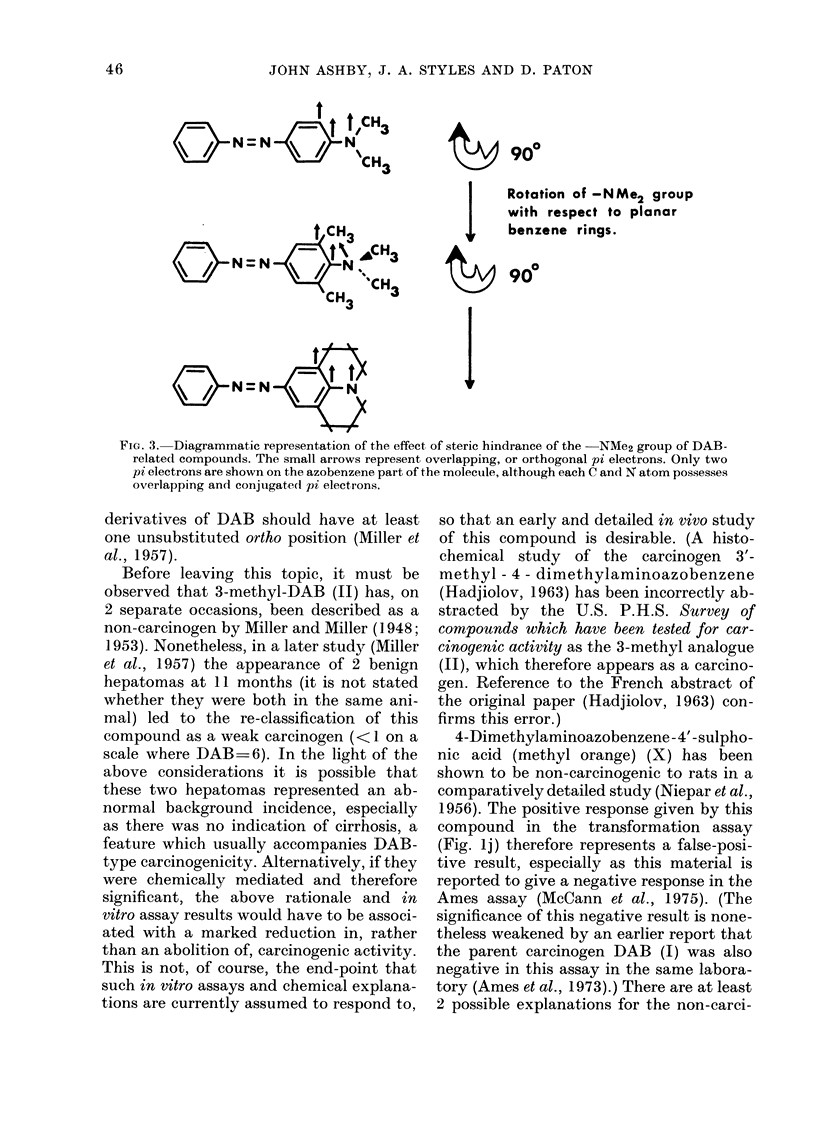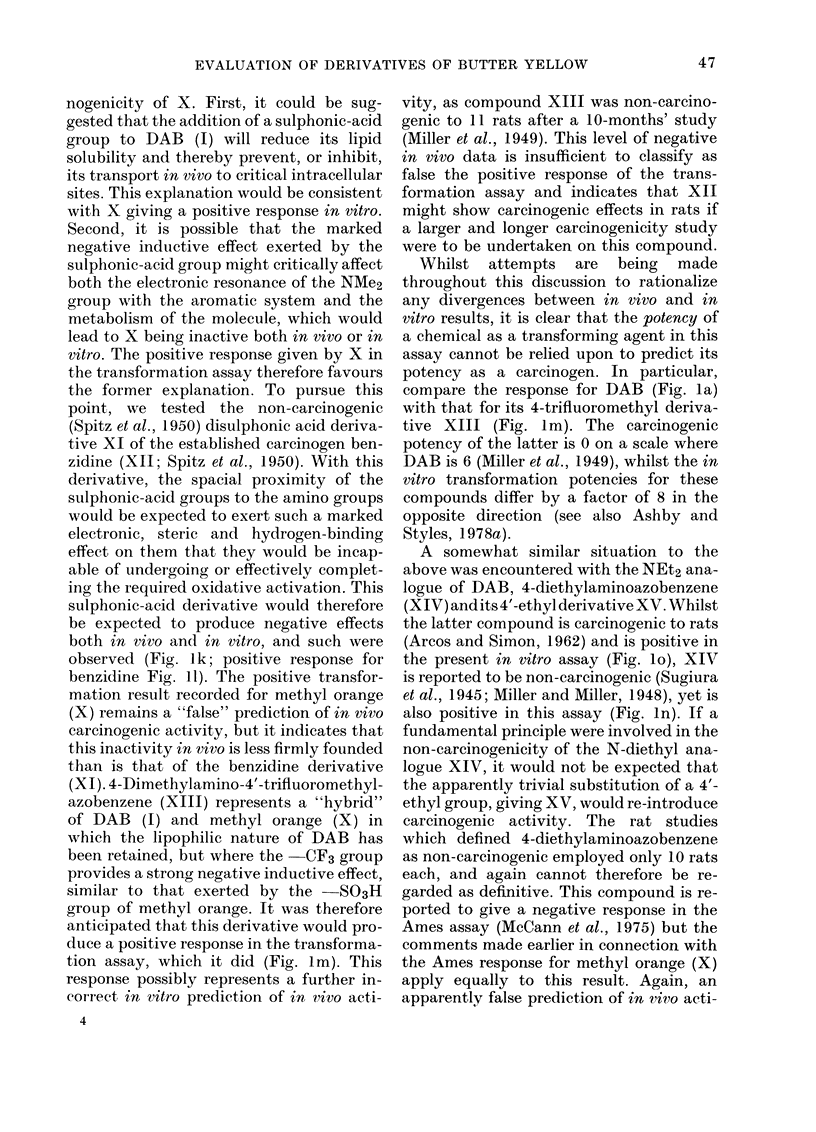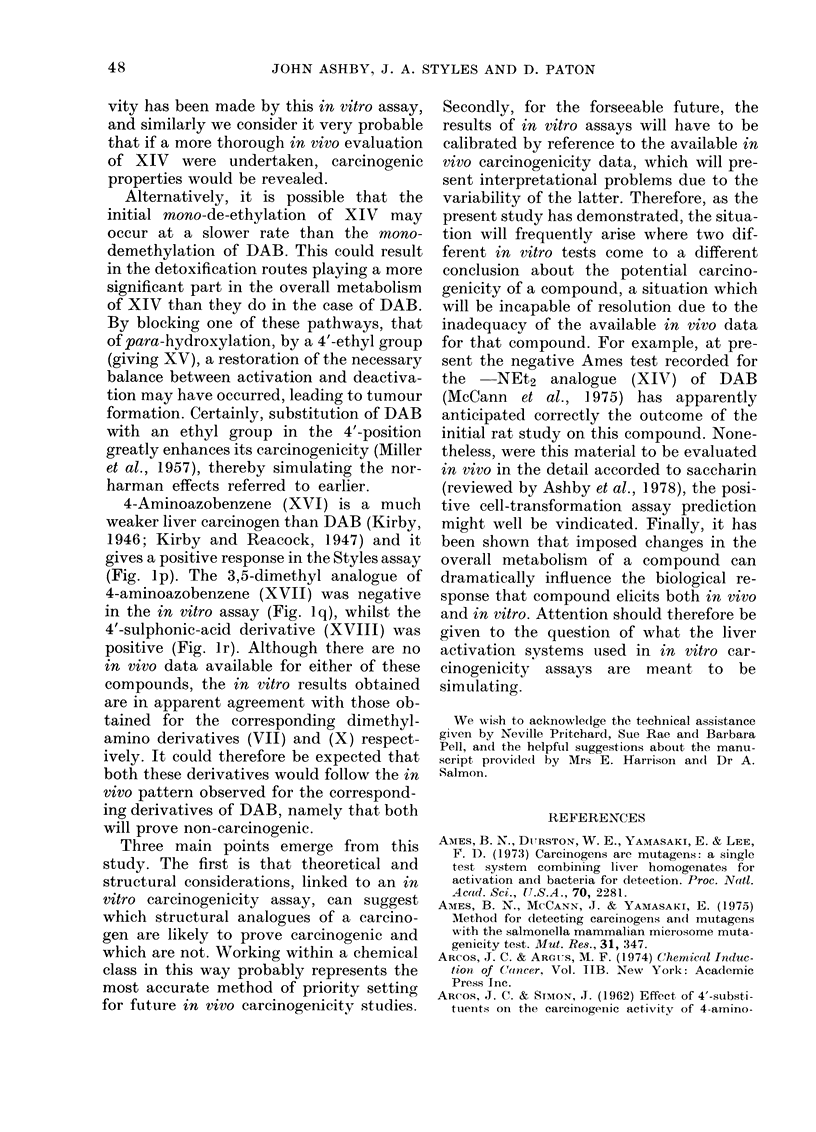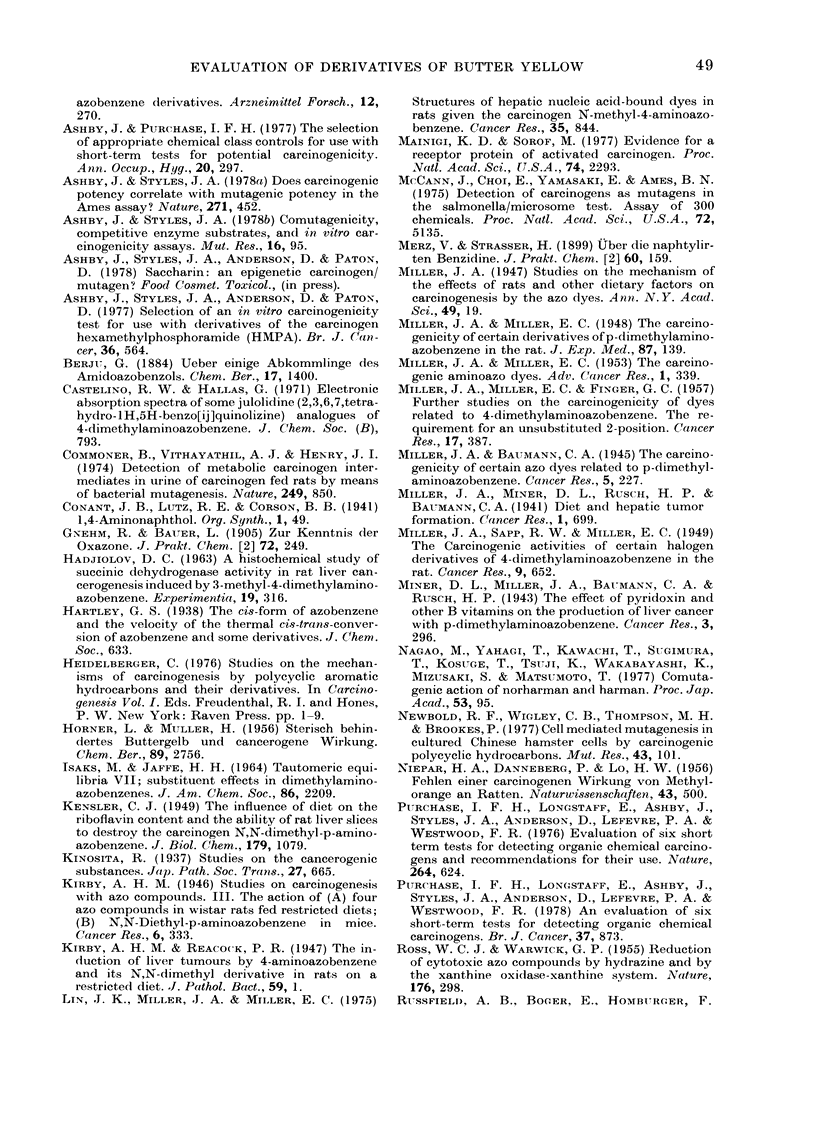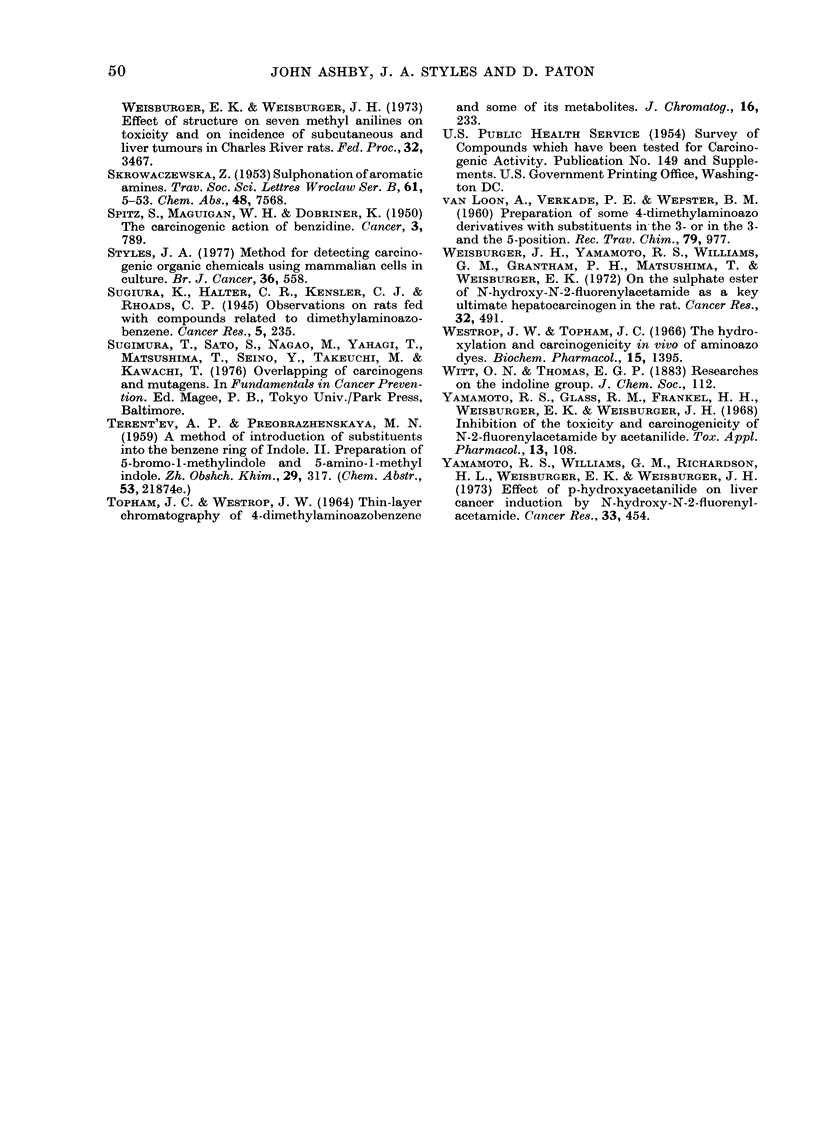# In vitro evaluation of some derivatives of the carcinogen butter yellow: implications for environmental screening.

**DOI:** 10.1038/bjc.1978.161

**Published:** 1978-07

**Authors:** J. Ashby, J. A. Styles, D. Paton

## Abstract

The rat-liver carcinogen 4-dimethylaminoazobenzene (Butter Yellow, DAB) and 12 of its structural analogues have been evaluated in a cell transformation assay. Eight of these analogues have already been tested for carcinogenicity in rats, whilst the remaining 4 are new or hitherto untested. Benzidine and its 3,3'-disulphonic acid derivative have also been evaluated. The in vitro results agree with long-term animal data for 8 compounds but disagree in finding DAB-4'-sulphonic acid, 4-trifluoromethyl-DAB and 4-diethylaminoazo-benzene positive. Possible reasons for these divergencies are discussed. It is concluded that 9-phenylazojulolidine and N-methyl-5-phenylazoindoline have carcinogenic potential and that 3,5-dimethyl-4-aminoazobenzene and 4-aminoazobenzene-4'-sulphonic acid are likely to prove non-carcinogenic. Addition of azobenzene to the in vitro assay medium increases the transforming potency of DAB 25-fold. It is suggested that it acts as a competitive substrate for one of the enzymes that detoxify DAB, and that this effect is related to that produced by norharman. Sulphonic-acid derivatives of established carcinogens are usually inactive. The basis of this effect has been investigated, and it is suggested that it can operate by two separate mechanisms. It has been established that this assay cannot be relied upon to predict the in vivo potency of a carcinogen. Consideration has been given to possible changes which could be made to the liver activation system (the S-9 mix) currently used in in vitro carcinogenicity assays, and a diagram is presented of the metabolic conversions of a compound which might lead to mutation or tumour formation. This enables the term potential carcinogen to be accurately defined, and indicates a possible difference between absolute non-carcinogens and compounds which fail to produce cancer in vivo.


*Br. J. Cancer* (1978) **38**, 34

### *IN VITRO* EVALUATION OF SOME DERIVATIVES OF THE CARCINOGEN BUTTER YELLOW: IMPLICATIONS FOR ENVIRONMENTAL SCREENING

JOHN ASHBY, J. A. STYLES AND D. PATON

*From Imperial Chemical Industries Limited, Central Toxicology Laboratory, Alderley Park, Macclesfield, Cheshire*

Received 9 March 1978   Accepted 30 March 1978

**Summary.—The rat-liver carcinogen 4-dimethylaminoazobenzene (Butter Yellow, DAB) and 12 of its structural analogues have been evaluated in a cell transformation assay. Eight of these analogues have already been tested for carcinogenicity in rats, whilst the remaining 4 are new or hitherto untested. Benzidine and its 3,3′-disulphonic acid derivative have also been evaluated.**

**The *in vitro* results agree with long-term animal data for 8 compounds but disagree in finding DAB-4′-sulphonic acid, 4-trifluoromethyl-DAB and 4-diethylaminoazobenzene positive. Possible reasons for these divergencies are discussed. It is concluded that 9-phenylazojulolidine and N-methyl-5-phenylazoindoline have carcinogenic potential and that 3,5-dimethyl-4-aminoazobenzene and 4-aminoazobenzene-4′-sulphonic acid are likely to prove non-carcinogenic.**

**Addition of azobenzene to the *in vitro* assay medium increases the transforming potency of DAB 25-fold. It is suggested that it acts as a competitive substrate for one of the enzymes that detoxify DAB, and that this effect is related to that produced by norharman.**

**Sulphonic-acid derivatives of established carcinogens are usually inactive. The basis of this effect has been investigated, and it is suggested that it can operate by two separate mechanisms.**

**It has been established that this assay cannot be relied upon to predict the *in vivo* potency of a carcinogen.**

**Consideration has been given to possible changes which could be made to the liver activation system (the S-9 mix) currently used in *in vitro* carcinogenicity assays, and a diagram is presented of the metabolic conversions of a compound which might lead to mutation or tumour formation. This enables the term *potential carcinogen* to be accurately defined, and indicates a possible difference between *absolute* non-carcinogens and compounds which *fail* to produce cancer *in vivo*.**

Butter Yellow (DAB; I), together with many of its substituted derivatives or functional analogues (U.S. P.H.S. Survey of Compounds which have been tested for carcinogenic activity; Arcos and Argus, 1974), form one of the most widely evaluated series of carcinogens, and they therefore present an interesting series in which to compare the findings of long-term animal carcinogenicity experiments with the predictions of possible carcinogenicity made by *in vitro* assays.

An *in vitro* carcinogenicity assay is usually judged by its ability to detect both carcinogens and non-carcinogens selected from a wide variety of structural classes (McCann *et al.*, 1975, Sugimura *et al.*, 1976; Purchase *et al.*, 1976, 1978) and although this is a necessary first step in the validation of a test it is not likely to define its strengths and weaknesses, especially if these properties are chemical-class related. The series of compounds based on DAB was selected for study because it was thought likely that some of the problems that might be expected to accompany the extrapolation of *in vitro* test predictions to the situation *in vivo* might thereby become apparent.

Butter Yellow was first identified as a carcinogen by Japanese workers in 1937 (Kinosita, 1937, and reviewed by Arcos and Argus, 1974) but initial attempts in America to repeat these observations were unsuccessful. This divergence of results was subsequently shown to be due to differences in the rat diets used in the two countries. By employing a polished rice (riboflavin-deficient) diet, the Japanese workers had unwittingly reduced the levels of azo-reductase enzymes (which are riboflavin-dependent) in the liver of the test animals. These enzymes have since been shown to be partly responsible for the deactivation of DAB; therefore, by reducing their levels, the Japanese workers had potentiated DAB-mediated tumour production. (This sequence of events has been reviewed in detail by Arcos and Argus, 1974.) A corollary to the above is that, had DAB been tested only under normal dietary conditions, it would have been recorded as a non-carcinogen. It could therefore be anticipated that the enzyme profile of the rat-liver metabolic system (the S-9 mix) used in an *in vitro* test would be both diet-dependent and critical in determining the response given by that test for DAB and its analogues. The second area of uncertainty and interest presented by these compounds flows from the fact that most of the carcinogenicity data available for analogues of DAB were generated by a standard test procedure which was limited by the restrictions that often only the liver of exposed animals was studied, and that most of the experiments were terminated between 9 and 11 months. Nonetheless, a tendency to regard such results as either clearly positive or negative has inevitably developed. These facts indicate that the results of an *in vitro* test might lead to a different conclusion regarding the possible carcinogenicity of an analogue of DAB than that indicated by the original animal study. It may be too simple to dismiss any such divergencies as *false in vitro* predictions; in fact it is suggested in this paper that the results of an appropriately controlled *in vitro* test can assist in the interpretation of an imperfect *in vivo* study.

The first step in the present study was to select the most appropriate test for this particular series of compounds from those which were available to us, namely, the salmonella reverse mutation assay of Ames *et al.* (1975) and the cell transformation assay of Styles (1977). Whilst DAB can produce a positive effect in the Ames assay, this response cannot be relied upon (Ames *et al.*, 1973; McCann *et al.*, 1975; Purchase *et al.*, 1978; Ashby and Purchase, 1977; Nagao *et al.*, 1977). Such uncertainty is important in a study such as the present one. A negative result for an analogue of DAB would be meaningless in the presence of a negative result for DAB itself, a self-imposed restraint which has led to many studies having to be repeated in our laboratory with other series of compounds. In contrast to the performance of the Ames assay, the Styles assay consistently found DAB positive in preliminary experiments. Therefore, by applying the test selection criteria described earlier (Ashby *et al.*, 1977) we adopted the cell transformation assay for this study.

All experiments with this assay were conducted using DAB as the chemical-class-positive control and with 3-methyl-4-dimethylaminoazobenzene (3-methyl-DAB, II) as negative control (Ashby and Purchase, 1977; for a discussion of this selection see later). Eleven compounds that had previously been evaluated in rats for carcinogenicity [Compounds I, II, VI, VII, X, XI, XII, XIII, XIV and XVI (see Chart)] were tested, together with 4 previously unevaluated derivatives of DAB [compounds VIII, IX, XVII and XVIII (see Chart)].

### MATERIALS AND METHODS

### *Chemicals*

The preparations of N-methyl-5-phenylazoindoline (IX) and benzidine-3,3′-disulphonic acid (XI) are here described in detail; the preparation of the remaining compounds is described briefly. The C, H and N content of each compound has been determined and, unless stated otherwise, is within 0·3% of the theoretical values. In addition, the NMR, IR and mass spectrum of each compound has been determined and is consistent with the structures shown (any exceptions are shown in square brackets). No significant impurities in any of the test compounds were detected by any of the above methods, or by TLC examination. NMR spectra were recorded either at 60 MHz using a Perkin-Elmer R-12 or Varian A-60 spectrometer, at 100 MHz using a Varian HA 100 (D) spectrometer, or at 90 MHz by Fourier transformation using a Bruker HX 90E spectrometer. Mass spectra were determined using either an AE1 MS9 or an MS 902 instrument ($M^+$ implies detection of the required mass ion).

4-*Dimethylaminoazobenzene* (*Butter Yellow, DAB, I*).—Supplied by B.D.H. Ltd, Dorset, and was further purified by recrystallization from cyclohexane in the presence of charcoal, m.p. 116°C (Berju, 1884: m.p. 117°C).

3-*Methyl*-4-*dimethylaminoazobenzene* (3-*methyl-DAB, II*).—Prepared by condensation of nitrosobenzene with 3-methyl-4-dimethylaminoaniline as reported by Van Loon *et al.* (1960). Chromatography on florisil, using chloroform as eluent, produced a red oil (70%) which could not be induced to crystallize.

*Azobenzene* (*VI*).—Purchased from B.D.H. Ltd, and appeared to be pure by all of the analytical techniques used, m.p. 67–68°C (Hartley, 1938: m.p. 68°C).

3,5 - *Dimethyl* - 4 - *dimethylaminoazobenzene* (*VII*).—Prepared by methylation of 3,5-dimethyl-4-aminoazobenzene (XVII) in Carius tubes at 75°C for 20 h using methyl iodide and sodium carbonate in a mixture of methanol and water as described by Horner and Müller (1956). Primary and secondary amine contaminants were removed by conversion to their acetyl derivatives, and the product purified by chromatography on silica gel, eluting with chloroform. The material so obtained was converted to its hydrochloride and crystallized from acetone (98%) m.p. 163–64°C (Horner and Muller, 1956: m.p. 151°C). [Theory for $C_{16}H_{20}N_3Cl$: C, 66·3; H, 6·9; N, 14·5. Found: C, 65·8; H, 7·0; N, 14·3%.]

9-*Phenylazojulolidine* (*VIII*).—Prepared by the method of Castelino and Hallas (1971) except that after removal of residual amines by steam distillation the product was purified by chromatography on florisil, eluting with toluene. After recrystallization from methanol containing a little petroleum ether (b.p. 60–80°C) the pure product had an m.p. of 83–84°C (Castelino and Hallas, 1971; m.p. 80–82°C).

*N-Methyl*-5-*phenylazoindoline* (*IX*).—Aniline was diazotized (Conant *et al.*, 1941) in the presence of 4 equivalents of HCl, and the diazonium solution added slowly to N-methylindoline in 4 equivalents of aqueous sodium acetate solution. After stirring for 3 h the orange-coloured precipitate was collected, washed with water and dried. It was purified by chromatography on florisil, eluting with chloroform, and was finally recrystallized from petroleum–ether (b.p. 60–80°C) to give red prisms (46%), m.p. 91–92°C. As this is a new compound, its full analytical data have been recorded. Theoretical for $C_{15}H_{15}N_3$ (237): C, 75·9; H, 6·3; N, 17·7. Found: C, 75·8; H, 6·4; N, 17·4%, $M^+$ 237; NMR (60 MHz, $CDCl_3$) 6·4 parts/$10^6$ (d) 1 H, H-7; 7·3–7·95 parts/$10^6$ (m) 7 H, remainder of aromatics; 2·78 parts/$10^6$ (s) 3H, N-methyl; 2·78–3·6 parts/$10^6$ (m) 4H, —$CH_2CH_2$—. Literature precedent for the electrophilic substitution of N-methylindoline taking place in the 5-position is given by Terent'ev and Preobrazhenskaya (1959) and is confirmed in this case by the presence in the NMR spectrum of the signal for H-7 (which would be expected to be the highest field aromatic proton) showing ortho-coupling.

4 - *Dimethylaminoazobenzene* - 4′ - *sulphonic acid, sodium salt* (*methyl orange, X*).—Obtained from B.D.H. Ltd and recrystallized from water in order to attain satisfactory analytical purity.

*Benzidine*-3,3′-*disulphonic acid* (*XI*).—Prepared by a modification of the method of Skrowaczewska (1953). Benzidine (11 g) and concentrated sulphuric acid (6·6 ml) were mixed together as thoroughly as possible before the start of the exothermic reaction, which was then allowed to proceed. When the reaction had subsided, diphenyl sulphone (20 g) was added. The vessel was evacuated (water-pump pressure) and heated for 7 h in an oil bath at 250°C. The mixture was allowed to cool and the solid mass broken up and finely ground in a mortar. The solid was treated with excess dilute aqueous NaOH solution and filtered. The residual solid was washed on the filter with NaOH solution and the combined filtrates acidified with dilute HCl. The precipitated product was collected, resuspended in water, filtered and dried (92%). The grey solid (m.p. >340°C) was soluble in cold DMSO but only sparingly soluble in other organic solvents. TLC (Merck silica gel GF, developed either in methanol or in chloroform/methanol 9:1) showed a less polar impurity at a level which was just detectable by u/v light. Theoretical for $C_{12}H_{12}N_2O_6S_2$ (344): C, 41·9; H, 3·5; N, 8·1. Found: C, 41·9; H, 3·7; N, 8·1%. At 310°C no $M^+$ 344 was observed, but a weak 265 (344-$SO_3$) and a strong 184 (265-$SO_3$) were seen. NMR (100 MHz, $DMSOd_6$) 7·2–7·9 parts/$10^6$ (m) aromatic; 8·55 parts/$10^6$ (s), $NH_2$, $SO_3H$.

*Benzidine* (*XII*).—Supplied by B.D.H. Ltd and was found to be of satisfactory purity as received, m.p. 128–29°C (Merz and Strasser, 1899; m.p. 128°C).

4 - *Dimethylamino* - 4′ - *trifluoromethylazobenzene* (*XIII*).—Obtained by diazonium coupling of 4-trifluoromethylaniline with N,N-dimethylaniline in the presence of sodium acetate. The product was collected, washed with water, dried, recrystallized from ethanol (m.p. 166–67°C) and then from benzene to yield golden-yellow plates m.p. 167–68°C (Isaks and Jaffe, 1964: m.p. 178–178·5°C). Despite the melting point remaining below that previously reported, this material appeared pure when judged by all other criteria. Further, its NMR spectrum clearly established *para-para* substitution.

4-*Diethylaminoazobenzene* (*XIV*).—Prepared by the method of Gnehm and Bauer (1905). After recrystallization from ethanol containing a little water, the product (44%) had an m.p. of 97–98°C (Gnehm and Bauer, 1905: m.p. 98–99°C). [Theoretical for $C_{16}H_{19}N_3$ hemihydrate: C, 73·3; H, 7·6; N, 16·0. Found: C, 73·0; H, 7·5; N, 16·0%.]

4 - *Diethylamino* - 4′ - *ethylazobenzene* (*XV*).—Prepared as described by Arcos and Simon (1962). After recrystallization from ethanol the product had an m.p. of 173–74°C (Arcos and Simon, 1962: m.p. 173·5–74·5°C).

4-*Aminoazobenzene* (*XVI*).—Supplied by I.C.I. Pharmaceuticals Division, Alderley Park, Macclesfield, Cheshire, and was further purified by recrystallization from toluene to give yellow needles of m.p. 125–26°C (Witt and Thomas, 1883: m.p. 125–26°C).

3,5-*Dimethyl*-4-*aminoazobenzene* (*XVII*).—Prepared by the method of Horner and Muller (1956) but the product was isolated as the violet-coloured hydrochloride, and recrystallized from n-butanol containing a few drops of 11 N HCl (20%) m.p. 196–97°C (no literature on m.p. available).

4 - *Aminoazobenzene* - 4′ - *sulphonic acid* (*XVIII*).—Supplied by I.C.I. Pharmaceuticals Division. It was found pure by all of the analytical techniques used and was tested as received. [Theoretical for $C_{12}H_{11}N_3O_3S + 1{\cdot}25\ H_2O$: C, 48·1; H, 4·5; N, 14·0. Found: C, 48·3; H, 4·3; N, 13·7%.]

*Cell-transformation test*.—The methods employed when testing a compound for potential carcinogenicity using growth of mammalian cells in semi-solid agar have been described in detail (Styles, 1977). A positive result is recorded when the transformation frequency per $10^6$ survivors at the $LD_{50}$ exceeds 5× the control frequency. The cells used in this study were BHK21/C13, which had a spontaneous transformation frequency of 50/$10^6$ survivors, or 10/$10^6$ survivors in a later clone. The solvent used in all experiments was DMSO (B.D.H. Chemicals Ltd, Poole, Dorset) and all experiments were conducted in the presence of Aroclor 1254-induced rat liver S-9 mix.

### RESULTS

Transformation frequencies (corrected to a theoretical $LD_0$) and cell survivals obtained after treatment of the cells with the following compounds listed below are shown in Fig. 1: 4-dimethylaminoazobenzene (I) (a, average of 7 experiments and b, using the new clone), 3-methyl-4-dimethylaminoazobenzene (II) (c, average of 3 experiments and d, using the new clone), azobenzene (VI) (e, using the new clone), a mixture of 4-dimethylaminoazobenzene (I) and azobenzene (VI), showing concentration of each compound mixed (f), 3,5-dimethyl-4-dimethylaminoazobenzene (VII) (g), 9-phenylazojulolidine (VIII) (h), N-methyl-5-phenylazoindoline (IX) (i), 4-dimethylaminoazobenzene-4′-sulphonic acid, sodium salt (methyl orange) (X) (j), benzidine-3,3′-disulphonic acid (XI) (k), benzidine (XII) (l, average of 5 experiments), 4-dimethylamino-4′-trifluoromethylazobenzene (XIII) (m), 4-diethylaminoazobenzene (XIV) (n), 4-diethylamino-4′-ethylazobenzene (XV) (o), 4-aminoazobenzene (XVI) (p), 3,5-dimethyl-4-aminoazobenzene (XVII) (q) and 4-aminoazobenzene - 4′ - sulphonic acid (XVIII) (r). The experiments were conducted in small groups using duplicate plates at each dose level, and on each occasion 4-dimethylaminoazobenzene (I) and its 3-methyl analogue (II) were used as the appropriate chemical-class-positive and -negative controls (Ashby and Purchase, 1977). In addition, all experiments were conducted in the presence of DMSO as a second negative control. Compounds I, VIII, IX, X, XII, XIII, XIV, XV, XVI and XVIII gave a positive response whilst compounds II, VI, VII, XI and XVII gave a negative response. Compounds I, II, VI and a mixture of compound I and VI were tested using a later clone with a spontaneous transformation frequency of 10. The results obtained with this clone (Figs 1b, d, e and f) are therefore scored as positive if transformation exceeds 50 transformants per $10^6$ survivors (reflected in a change in the position of the horizontal dotted line in these figures as compared with the other figures).

### DISCUSSION

The carcinogenic activation of DAB has been suggested as proceeding *via* oxidative *mono*-demethylation accompanied by N-oxidation, yielding the N-hydroxy derivative (III) of 4-methylaminoazobenzene (Scheme) (Lin *et al.*, 1975, reviewed by Arcos and Argus, 1974). Subsequent esterification of this N-hydroxy derivative is thought to lead to the formation of an electrophilic species (formally suggested to be a nitrenium ion (Lin *et al.*, 1975)) which reacts with guanosine in the 8-position (and perhaps at other sites on DNA).

It has been suggested that intracellular transport of this activated species occurs *via* a receptor protein (Mainigi and Sorof, 1977). The active ester formed from compounds such as III has generally been assumed to be the sulphate derivative, but this idea has recently been questioned in the case of the metabolic activation of the carcinogen 2-acetylaminofluorene. In this

SCHEME

Fig. 1.—Survival and transformation of BHK cells treated with Compounds I (a and b), II (c and d), VI (e), I+VI (f), VII (g), VIII (h), IX (i), X (j), XI (k), XII (l), XIII (m), XIV (n), XV (o), XVI (p), XVII (q) and XVIII (r). Dashed lines represent (above) 50% survival (and $LD_{50}$) and (below) 250 transformants (or 50 transformants for clone shown in Figs b, d, e and f) per $10^6$ survivors (*i.e.* 5 × control frequency).

FIG. 1(a–f).

% survivors transformants per $10^6$ survivors

μg/ml

N=N

$CH_3$

a b c d e f

0.025

0.25

2.5

25

250

2500

1,100

10,000

9,000

FIG. 1(g–l)

% survivors

100

50

0

transformants per $10^6$ survivors

1,100

900

700

500

300

100

0

0.025

0.25

2.5

25

250

μg/ml g h i j k

2500

Induced transformants per $10^6$ survivors l

FIG. 1(m–r)

% survivors

100

50

0

1,100

900

700

500

300

100

transformants per $10^6$ survivors

0

0.025

0.25

2.5

25

250

μg/ml m n o p q r

### CHART

(I)

(II)

(VI)

(VII)

(VIII)

(IX)

(X)

(XI)

(XII)

(XIII)

(XIV)

(XV)

(XVI)

(XVII)

(XVIII)

(XIX)

case, the formation of an acetate ester has been postulated (Yamamoto *et al.*, 1968; Weisburger *et al.*, 1972; Yamamoto *et al.*, 1973) and this might also apply to other carcinogens such as DAB.

There is also evidence for the existence of two competitive, metabolic detoxification routes for DAB and its derivatives. The first involves cleavage of the compound at the azo-linkage *via* azoreductase enzymes, yielding, in the case of DAB, the anilines IV and V. The second involves ring hydroxylation followed by conjugation (Lin *et al.*, 1974; Commoner *et al.*, 1974; Topham and Westrop, 1964; Westrop and Topham, 1966) (Scheme). Evidence that the azoreductase pathway results in the detoxification of DAB is afforded by the observations that both IV and V are non-carcinogenic under the same test conditions as those in which DAB is carcinogenic (Miller and Baumann, 1945; Miller and Miller, 1948; Miller *et al.*, 1957), and by the observation that rats treated with DAB develop a higher incidence of liver tumours when the level of their liver azoreductase enzymes is artificially depressed by diet manipulation (Miller *et al.*, 1941; Miner *et al.*, 1943; Miller, 1947; Kensler, 1949).

Clearly, variations in the relative contribution made by these 3 metabolic pathways could give rise to variations in both the *in vivo* and *in vitro* responses to DAB and its analogues. In particular, under conditions where the rate of azo-reductive or ring-oxidative deactivation of DAB critically exceeded the rate of whole-molecule N-oxidation, a reduced, or perhaps abolished, biological response might be expected. The early problems encountered when attempting to obtain a reproducible carcinogenic effect for DAB (reviewed by Arcos and Argus, 1974) and the current problems in obtaining a reproducible response for this compound in the salmonella assay may therefore be related. The reproducibility of the cell transformation assay response for DAB (I) (Fig. 1a and b) may be due to augmentation of the metabolic activity of the rat liver S-9 mix by hamster and other rodent cells' innate capacity for oxidative metabolism (Newbold *et al.*, 1977; Heidelberger, 1976). Nonetheless, these cells are incapable of completing the activation of DAB by themselves, as evidenced by the negative response produced by this compound either in the absence of rat liver S-9 mix or in the presence of uninduced liver homogenate. These negative results may be due to an inability to esterify any N-hydroxy intermediate (III) that might be formed by the cells (cell toxicity is observed). Alternatively, stabilization of metabolically activated DAB by appropriate receptor proteins (Mainigi and Sorof, 1977) may be occurring in the BHK cells but not in the salmonella, thereby increasing the relative half-life and selective reactivity of such species in the cell-based test.

In an attempt to demonstrate the importance of metabolic detoxification pathways to the response given by an *in vitro* test, we tested DAB in the cell-transformation assay in the presence of azobenzene (VI). This material, whilst being non-carcinogenic to rats (Spitz *et al.*, 1950) and negative in the present assay (Fig. 1e) was intended as a substrate for both the C-hydroxylase and azoreductase enzymes of the S-9 mix. It was expected that by testing DAB in the presence of azobenzene the contribution made by the critical activation pathway for DAB would be increased with respect to those pathways acting against the production of a DNA-active species. The enhanced response obtained is shown in Fig. 1(f), and serves to underline the variability in *in vitro* test response to be expected with changes in the S-9 mix or overall *in vitro* metabolism (Ashby and Styles 1978*a*, *b*). These effects closely parallel those produced by norharman with DAB in the Ames assay (Nagao *et al.*, 1977) and those produced by 4′ethylation of DAB, to be discussed later. These observations may also cast some light on the fact that most derivatives of DAB carrying a substituent *ortho* to the azo bridge are non-carcinogenic (reviewed by Arcos and Argus, 1974). In a related series of azobenzene alkylating agents, Ross and Warwick (1955) have shown that those derivatives bearing substituents *ortho* to the azo linkage undergo accelerated reductive cleavage of the azo group. It may therefore be an increased rate of azoreductase-mediated detoxification which renders the corresponding analogues of DAB non-carcinogenic. The potential of these derivatives of DAB to cause cancer may still be present, but their ability to express this potential may have been metabolically limited to zero. A major exception to this hypothesis is provided by 2′,3′-dimethyl-4-dimethylaminoazobenzene, which is a potent liver carcinogen. The inexplicability of this high activity has been commented on already (Arcos and Argus, 1974, p. 160) but can, nonetheless, perhaps be explained in the light of the above considerations. With this compound, accelerated reductive cleavage will lead to the formation in the liver of 2,3-dimethylaniline. This compound, although of unknown carcinogenicity, is likely itself to be a liver carcinogen (Russfield *et al.*, 1973). Therefore in this case cleavage of the azo link may lead to the *in situ* formation of another liver carcinogen.

The above reasoning leads to the hypothesis that many compounds may be chemically equipped, and theoretically able to cause cancer under individually optimized metabolic circumstances, but only a proportion of these may be capable of actually inducing tumours under the metabolic conditions of an *in vivo* study. If this is true, a dilemma is posed by the possibility that *in vitro* carcinogenicity assays may sometimes detect the *potential* rather than the *ability in vivo* of a chemical to cause cancer. As the number of compounds in the former category may be significantly larger than those in the latter, some attention should be given to what is inferred from the results of such *in vitro* assays. In particular, the following question should be answered: are carcinogen-screening programmes designed to protect the majority of a population from exposure to easily demonstrable animal carcinogens, or are they also to be used to protect all metabolically idiosyncratic minorities of a population from each and every possible carcinogen? (The metabolic differences of these subgroups may be environmentally or genetically determined.) The existence of such sub-groups is probably evidenced by the non-uniform incidence of tumours generally observed when either animals or humans are exposed to chemical carcinogens. The answer to the above question will determine whether the enzyme profile of the S-9 liver fraction used in *in vitro* assays should be regulated, as far as is possible, to that encountered by a chemical in the liver of an average man, or if it is to be individually optimized, perhaps as a "cocktail" of individually purified enzymes, to give the maximum chance of obtaining a positive response for each compound. The above considerations are shown diagrammatically in Fig. 2, which enables the term *potential carcinogen* to be accurately defined, and which indicates that there are probably 2 classes of non-carcinogens recorded in the literature. The first, a group of *absolute non-carcinogens* and the second, compounds which are *potential carcinogens* but which have so far given only a *non-carcinogenic effect in vivo*. Chemicals within the latter group could possibly be induced to produce tumours by the appropriate choice of animal species and strain, or diet etc. These arguments cast doubt on the historical concept of *absolute* carcinogens and non-carcinogens, but this should not be abandoned too readily, at least not until an alternative ground-rock can be found upon which to base decisions concerning the potential human hazard presented by exposure to a given chemical.

3,5-Dimethyl-DAB (VII) was first synthesized by Horner and Muller (1956) as a possible non-carcinogenic analogue of DAB (I). In this compound, the two *ortho* methyl groups interact sterically with the 4-dimethylamino function and thereby prevent the nitrogen base-pair of electrons from conjugating with the aromatic ring system. This interaction is amply demon-

FIG. 2.—Diagrammatic representation of the moderating effects produced by competitive metabolic pathways on the carcinogenicity of a compound. The starting compound is assumed not to be a direct-acting carcinogen. In such cases the compound would be placed directly in the central box. The position of the indicator in each dial could affect both the *in vivo* and *in vitro* response given by a compound. The exact indicator positions will be influenced by the diet, sex, age, species and strain of the test animal, and dose levels employed in an *in vivo* study, the method of induction, preparation and storage of the liver S-9 mix used in an *in vitro* assay and the presence of competitive enzyme substrates, both *in vivo* and *in vitro*.

strated by reference to molecular models and by the colour and u.v. spectrum of VII when compared with DAB (Horner and Muller, 1956). Horner and Muller anticipated that this steric restraint, which effectively transforms VII into an analogue of azobenzene (VI) rather than of DAB (I), would also convert it into a non-carcinogen. The carcinogenicity experiment conducted on VII by Druckrey (Horner and Muller, 1956) confirms this prediction, as does the negative response it gave in the cell-transformation assay (Fig. 1g). This rationale would apply equally to 3-methyl-DAB (II), the presence of a second *ortho* methyl group being redundant to the above steric explanation, and again the transformation assay (Fig. 1c and d) and 2 carcinogenicity experiments in rats confirm this prediction (see later). The inactivity of compounds II and VII is probably due to the abolition (or critical reduction) of oxidative demethylation by steric or electronic factors. The carcinogenicity of 3-methyl-4-methylaminoazobenzene (XIX; Miller and Miller, 1948), the sterically unrestricted *mono*-demethylation product of (II), indicates that this "block" occurs early in the metabolic sequence. An indication that the sterically enforced deconjugation of the substituent nitrogen atom from the aromatic system is responsible for the inactivity of both II and VII (Fig. 3) is afforded by the observations that both the julolidine derivative (VIII) and the indoline derivative (IX) are positive in the transformation assay (Figs. 1h and i, respectively). Compound VIII represents a close derivative of Compound VII in which planarity between the nitrogen substituent and the aromatic system has been restored, and with it the ability of the nitrogen lone-pair of electrons to conjugate with the aromatic system (Fig. 3). The indoline (IX) is a similar analogue of Compound II. Both the julolidine derivative (VIII) and the indoline derivative (IX) must therefore be regarded as potentially carcinogenic compounds. The *in vitro* activity of the julolidine derivative (VIII) is at variance with the idea that, in order to show activity,

FIG. 3.—Diagrammatic representation of the effect of steric hindrance of the —$NMe_2$ group of DAB-related compounds. The small arrows represent overlapping, or orthogonal *pi* electrons. Only two *pi* electrons are shown on the azobenzene part of the molecule, although each C and N atom possesses overlapping and conjugated *pi* electrons.

derivatives of DAB should have at least one unsubstituted *ortho* position (Miller *et al.*, 1957).

Before leaving this topic, it must be observed that 3-methyl-DAB (II) has, on 2 separate occasions, been described as a non-carcinogen by Miller and Miller (1948; 1953). Nonetheless, in a later study (Miller *et al.*, 1957) the appearance of 2 benign hepatomas at 11 months (it is not stated whether they were both in the same animal) led to the re-classification of this compound as a weak carcinogen (< 1 on a scale where DAB = 6). In the light of the above considerations it is possible that these two hepatomas represented an abnormal background incidence, especially as there was no indication of cirrhosis, a feature which usually accompanies DAB-type carcinogenicity. Alternatively, if they were chemically mediated and therefore significant, the above rationale and *in vitro* assay results would have to be associated with a marked reduction in, rather than an abolition of, carcinogenic activity. This is not, of course, the end-point that such *in vitro* assays and chemical explanations are currently assumed to respond to, so that an early and detailed *in vivo* study of this compound is desirable. (A histochemical study of the carcinogen 3′-methyl-4-dimethylaminoazobenzene (Hadjiolov, 1963) has been incorrectly abstracted by the U.S. P.H.S. *Survey of compounds which have been tested for carcinogenic activity* as the 3-methyl analogue (II), which therefore appears as a carcinogen. Reference to the French abstract of the original paper (Hadjiolov, 1963) confirms this error.)

4-Dimethylaminoazobenzene-4′-sulphonic acid (methyl orange) (X) has been shown to be non-carcinogenic to rats in a comparatively detailed study (Niepar *et al.*, 1956). The positive response given by this compound in the transformation assay (Fig. 1j) therefore represents a false-positive result, especially as this material is reported to give a negative response in the Ames assay (McCann *et al.*, 1975). (The significance of this negative result is nonetheless weakened by an earlier report that the parent carcinogen DAB (I) was also negative in this assay in the same laboratory (Ames *et al.*, 1973).) There are at least 2 possible explanations for the non-carcinogenicity of X. First, it could be suggested that the addition of a sulphonic-acid group to DAB (I) will reduce its lipid solubility and thereby prevent, or inhibit, its transport *in vivo* to critical intracellular sites. This explanation would be consistent with X giving a positive response *in vitro*. Second, it is possible that the marked negative inductive effect exerted by the sulphonic-acid group might critically affect both the electronic resonance of the $NMe_2$ group with the aromatic system and the metabolism of the molecule, which would lead to X being inactive both *in vivo* or *in vitro*. The positive response given by X in the transformation assay therefore favours the former explanation. To pursue this point, we tested the non-carcinogenic (Spitz *et al.*, 1950) disulphonic acid derivative XI of the established carcinogen benzidine (XII; Spitz *et al.*, 1950). With this derivative, the spacial proximity of the sulphonic-acid groups to the amino groups would be expected to exert such a marked electronic, steric and hydrogen-binding effect on them that they would be incapable of undergoing or effectively completing the required oxidative activation. This sulphonic-acid derivative would therefore be expected to produce negative effects both *in vivo* and *in vitro*, and such were observed (Fig. 1k; positive response for benzidine Fig. 1l). The positive transformation result recorded for methyl orange (X) remains a "false" prediction of *in vivo* carcinogenic activity, but it indicates that this inactivity *in vivo* is less firmly founded than is that of the benzidine derivative (XI). 4-Dimethylamino-4′-trifluoromethylazobenzene (XIII) represents a "hybrid" of DAB (I) and methyl orange (X) in which the lipophilic nature of DAB has been retained, but where the —$CF_3$ group provides a strong negative inductive effect, similar to that exerted by the —$SO_3H$ group of methyl orange. It was therefore anticipated that this derivative would produce a positive response in the transformation assay, which it did (Fig. 1m). This response possibly represents a further incorrect *in vitro* prediction of *in vivo* activity, as compound XIII was non-carcinogenic to 11 rats after a 10-months' study (Miller *et al.*, 1949). This level of negative *in vivo* data is insufficient to classify as false the positive response of the transformation assay and indicates that XII might show carcinogenic effects in rats if a larger and longer carcinogenicity study were to be undertaken on this compound.

Whilst attempts are being made throughout this discussion to rationalize any divergences between *in vivo* and *in vitro* results, it is clear that the *potency* of a chemical as a transforming agent in this assay cannot be relied upon to predict its potency as a carcinogen. In particular, compare the response for DAB (Fig. 1a) with that for its 4-trifluoromethyl derivative XIII (Fig. 1m). The carcinogenic potency of the latter is 0 on a scale where DAB is 6 (Miller *et al.*, 1949), whilst the *in vitro* transformation potencies for these compounds differ by a factor of 8 in the opposite direction (see also Ashby and Styles, 1978*a*).

A somewhat similar situation to the above was encountered with the $NEt_2$ analogue of DAB, 4-diethylaminoazobenzene (XIV) and its 4′-ethyl derivative XV. Whilst the latter compound is carcinogenic to rats (Arcos and Simon, 1962) and is positive in the present *in vitro* assay (Fig. 1o), XIV is reported to be non-carcinogenic (Sugiura *et al.*, 1945; Miller and Miller, 1948), yet is also positive in this assay (Fig. 1n). If a fundamental principle were involved in the non-carcinogenicity of the N-diethyl analogue XIV, it would not be expected that the apparently trivial substitution of a 4′-ethyl group, giving XV, would re-introduce carcinogenic activity. The rat studies which defined 4-diethylaminoazobenzene as non-carcinogenic employed only 10 rats each, and again cannot therefore be regarded as definitive. This compound is reported to give a negative response in the Ames assay (McCann *et al.*, 1975) but the comments made earlier in connection with the Ames response for methyl orange (X) apply equally to this result. Again, an apparently false prediction of *in vivo* activity has been made by this *in vitro* assay, and similarly we consider it very probable that if a more thorough *in vivo* evaluation of XIV were undertaken, carcinogenic properties would be revealed.

Alternatively, it is possible that the initial *mono*-de-ethylation of XIV may occur at a slower rate than the *mono*-demethylation of DAB. This could result in the detoxification routes playing a more significant part in the overall metabolism of XIV than they do in the case of DAB. By blocking one of these pathways, that of *para*-hydroxylation, by a 4′-ethyl group (giving XV), a restoration of the necessary balance between activation and deactivation may have occurred, leading to tumour formation. Certainly, substitution of DAB with an ethyl group in the 4′-position greatly enhances its carcinogenicity (Miller *et al.*, 1957), thereby simulating the nor-harman effects referred to earlier.

4-Aminoazobenzene (XVI) is a much weaker liver carcinogen than DAB (Kirby, 1946; Kirby and Reacock, 1947) and it gives a positive response in the Styles assay (Fig. 1p). The 3,5-dimethyl analogue of 4-aminoazobenzene (XVII) was negative in the *in vitro* assay (Fig. 1q), whilst the 4′-sulphonic-acid derivative (XVIII) was positive (Fig. 1r). Although there are no *in vivo* data available for either of these compounds, the *in vitro* results obtained are in apparent agreement with those obtained for the corresponding dimethyl-amino derivatives (VII) and (X) respectively. It could therefore be expected that both these derivatives would follow the *in vivo* pattern observed for the corresponding derivatives of DAB, namely that both will prove non-carcinogenic.

Three main points emerge from this study. The first is that theoretical and structural considerations, linked to an *in vitro* carcinogenicity assay, can suggest which structural analogues of a carcinogen are likely to prove carcinogenic and which are not. Working within a chemical class in this way probably represents the most accurate method of priority setting for future *in vivo* carcinogenicity studies. Secondly, for the forseeable future, the results of *in vitro* assays will have to be calibrated by reference to the available *in vivo* carcinogenicity data, which will present interpretational problems due to the variability of the latter. Therefore, as the present study has demonstrated, the situation will frequently arise where two different *in vitro* tests come to a different conclusion about the potential carcinogenicity of a compound, a situation which will be incapable of resolution due to the inadequacy of the available *in vivo* data for that compound. For example, at present the negative Ames test recorded for the —$NEt_2$ analogue (XIV) of DAB (McCann *et al.*, 1975) has apparently anticipated correctly the outcome of the initial rat study on this compound. Nonetheless, were this material to be evaluated *in vivo* in the detail accorded to saccharin (reviewed by Ashby *et al.*, 1978), the positive cell-transformation assay prediction might well be vindicated. Finally, it has been shown that imposed changes in the overall metabolism of a compound can dramatically influence the biological response that compound elicits both *in vivo* and *in vitro*. Attention should therefore be given to the question of what the liver activation systems used in *in vitro* carcinogenicity assays are meant to be simulating.

We wish to acknowledge the technical assistance given by Neville Pritchard, Sue Rae and Barbara Pell, and the helpful suggestions about the manuscript provided by Mrs E. Harrison and Dr A. Salmon.

### REFERENCES

AMES, B. N., DURSTON, W. E., YAMASAKI, E. & LEE, F. D. (1973) Carcinogens are mutagens: a single test system combining liver homogenates for activation and bacteria for detection. *Proc. Natl. Acad. Sci., U.S.A.*, **70**, 2281.

AMES, B. N., MCCANN, J. & YAMASAKI, E. (1975) Method for detecting carcinogens and mutagens with the salmonella mammalian microsome mutagenicity test. *Mut. Res.*, **31**, 347.

ARCOS, J. C. & ARGUS, M. F. (1974) *Chemical Induction of Cancer*, Vol. IIB. New York: Academic Press Inc.

ARCOS, J. C. & SIMON, J. (1962) Effect of 4′-substituents on the carcinogenic activity of 4-aminoazobenzene derivatives. *Arzneimittel Forsch.*, **12**, 270.

ASHBY, J. & PURCHASE, I. F. H. (1977) The selection of appropriate chemical class controls for use with short-term tests for potential carcinogenicity. *Ann. Occup., Hyg.*, **20**, 297.

ASHBY, J. & STYLES, J. A. (1978*a*) Does carcinogenic potency correlate with mutagenic potency in the Ames assay? *Nature*, **271**, 452.

ASHBY, J. & STYLES, J. A. (1978*b*) Comutagenicity, competitive enzyme substrates, and *in vitro* carcinogenicity assays. *Mut. Res.*, **16**, 95.

ASHBY, J., STYLES, J. A., ANDERSON, D. & PATON, D. (1978) Saccharin: an epigenetic carcinogen/mutagen? *Food Cosmet. Toxicol.*, (in press).

ASHBY, J., STYLES, J. A., ANDERSON, D. & PATON, D. (1977) Selection of an *in vitro* carcinogenicity test for use with derivatives of the carcinogen hexamethylphosphoramide (HMPA). *Br. J. Cancer*, **36**, 564.

BERJU, G. (1884) Ueber einige Abkommlinge des Amidoazobenzols. *Chem. Ber.*, **17**, 1400.

CASTELINO, R. W. & HALLAS, G. (1971) Electronic absorption spectra of some julolidine (2,3,6,7,tetrahydro-1H,5H-benzo[ij]quinolizine) analogues of 4-dimethylaminoazobenzene. *J. Chem. Soc.* (*B*), 793.

COMMONER, B., VITHAYATHIL, A. J. & HENRY, J. I. (1974) Detection of metabolic carcinogen intermediates in urine of carcinogen fed rats by means of bacterial mutagenesis. *Nature*, **249**, 850.

CONANT, J. B., LUTZ, R. E. & CORSON, B. B. (1941) 1,4-Aminonaphthol. *Org. Synth.*, **1**, 49.

GNEHM, R. & BAUER, L. (1905) Zur Kenntnis der Oxazone. *J. Prakt. Chem.* [2] **72**, 249.

HADJIOLOV, D. C. (1963) A histochemical study of succinic dehydrogenase activity in rat liver cancerogenesis induced by 3-methyl-4-dimethylaminoazobenzene. *Experimentia*, **19**, 316.

HARTLEY, G. S. (1938) The *cis*-form of azobenzene and the velocity of the thermal *cis-trans*-conversion of azobenzene and some derivatives. *J. Chem. Soc.*, 633.

HEIDELBERGER, C. (1976) Studies on the mechanisms of carcinogenesis by polycyclic aromatic hydrocarbons and their derivatives. In *Carcinogenesis Vol. I.* Eds. Freudenthal, R. I. and Hones, P. W. New York: Raven Press. pp. 1–9.

HORNER, L. & MULLER, H. (1956) Sterisch behindertes Buttergelb und cancerogene Wirkung. *Chem. Ber.*, **89**, 2756.

ISAKS, M. & JAFFE, H. H. (1964) Tautomeric equilibria VII; substituent effects in dimethylaminoazobenzenes. *J. Am. Chem. Soc.*, **86**, 2209.

KENSLER, C. J. (1949) The influence of diet on the riboflavin content and the ability of rat liver slices to destroy the carcinogen N,N-dimethyl-p-aminoazobenzene. *J. Biol. Chem.*, **179**, 1079.

KINOSITA, R. (1937) Studies on the cancerogenic substances. *Jap. Path. Soc. Trans.*, **27**, 665.

KIRBY, A. H. M. (1946) Studies on carcinogenesis with azo compounds. III. The action of (A) four azo compounds in wistar rats fed restricted diets; (B) N,N-Diethyl-p-aminoazobenzene in mice. *Cancer Res.*, **6**, 333.

KIRBY, A. H. M. & REACOCK, P. R. (1947) The induction of liver tumours by 4-aminoazobenzene and its N,N-dimethyl derivative in rats on a restricted diet. *J. Pathol. Bact.*, **59**, 1.

LIN, J. K., MILLER, J. A. & MILLER, E. C. (1975) Structures of hepatic nucleic acid-bound dyes in rats given the carcinogen N-methyl-4-aminoazobenzene. *Cancer Res.*, **35**, 844.

MAINIGI, K. D. & SOROF, M. (1977) Evidence for a receptor protein of activated carcinogen. *Proc. Natl. Acad. Sci., U.S.A.*, **74**, 2293.

McCANN, J., CHOI, E., YAMASAKI, E. & AMES, B. N. (1975) Detection of carcinogens as mutagens in the salmonella/microsome test. Assay of 300 chemicals. *Proc. Natl. Acad. Sci., U.S.A.*, **72**, 5135.

MERZ, V. & STRASSER, H. (1899) Über die naphtylirten Benzidine. *J. Prakt. Chem.* [2] **60**, 159.

MILLER, J. A. (1947) Studies on the mechanism of the effects of rats and other dietary factors on carcinogenesis by the azo dyes. *Ann. N.Y. Acad. Sci.*, **49**, 19.

MILLER, J. A. & MILLER, E. C. (1948) The carcinogenicity of certain derivatives of p-dimethylaminoazobenzene in the rat. *J. Exp. Med.*, **87**, 139.

MILLER, J. A. & MILLER, E. C. (1953) The carcinogenic aminoazo dyes. *Adv. Cancer Res.*, **1**, 339.

MILLER, J. A., MILLER, E. C. & FINGER, G. C. (1957) Further studies on the carcinogenicity of dyes related to 4-dimethylaminoazobenzene. The requirement for an unsubstituted 2-position. *Cancer Res.*, **17**, 387.

MILLER, J. A. & BAUMANN, C. A. (1945) The carcinogenicity of certain azo dyes related to p-dimethylaminoazobenzene. *Cancer Res.*, **5**, 227.

MILLER, J. A., MINER, D. L., RUSCH, H. P. & BAUMANN, C. A. (1941) Diet and hepatic tumor formation. *Cancer Res.*, **1**, 699.

MILLER, J. A., SAPP, R. W. & MILLER, E. C. (1949) The Carcinogenic activities of certain halogen derivatives of 4-dimethylaminoazobenzene in the rat. *Cancer Res.*, **9**, 652.

MINER, D. L., MILLER, J. A., BAUMANN, C. A. & RUSCH, H. P. (1943) The effect of pyridoxin and other B vitamins on the production of liver cancer with p-dimethylaminoazobenzene. *Cancer Res.*, **3**, 296.

NAGAO, M., YAHAGI, T., KAWACHI, T., SUGIMURA, T., KOSUGE, T., TSUJI, K., WAKABAYASHI, K., MIZUSAKI, S. & MATSUMOTO, T. (1977) Comutagenic action of norharman and harman. *Proc. Jap. Acad.*, **53**, 95.

NEWBOLD, R. F., WIGLEY, C. B., THOMPSON, M. H. & BROOKES, P. (1977) Cell mediated mutagenesis in cultured Chinese hamster cells by carcinogenic polycyclic hydrocarbons. *Mut. Res.*, **43**, 101.

NIEPAR, H. A., DANNEBERG, P. & LO, H. W. (1956) Fehlen einer carcinogenen Wirkung von Methylorange an Ratten. *Naturwissenschaften*, **43**, 500.

PURCHASE, I. F. H., LONGSTAFF, E., ASHBY, J., STYLES, J. A., ANDERSON, D., LEFEVRE, P. A. & WESTWOOD, F. R. (1976) Evaluation of six short term tests for detecting organic chemical carcinogens and recommendations for their use. *Nature*, **264**, 624.

PURCHASE, I. F. H., LONGSTAFF, E., ASHBY, J., STYLES, J. A., ANDERSON, D., LEFEVRE, P. A. & WESTWOOD, F. R. (1978) An evaluation of six short-term tests for detecting organic chemical carcinogens. *Br. J. Cancer*, **37**, 873.

ROSS, W. C. J. & WARWICK, G. P. (1955) Reduction of cytotoxic azo compounds by hydrazine and by the xanthine oxidase-xanthine system. *Nature*, **176**, 298.

RUSSFIELD, A. B., BOGER, E., HOMBURGER, F.

WEISBURGER, E. K. & WEISBURGER, J. H. (1973) Effect of structure on seven methyl anilines on toxicity and on incidence of subcutaneous and liver tumours in Charles River rats. *Fed. Proc.*, **32**, 3467.

SKROWACZEWSKA, Z. (1953) Sulphonation of aromatic amines. *Trav. Soc. Sci. Lettres Wroclaw Ser. B*, **61**, 5–53. *Chem. Abs.*, **48**, 7568.

SPITZ, S., MAGUIGAN, W. H. & DOBRINER, K. (1950) The carcinogenic action of benzidine. *Cancer*, **3**, 789.

STYLES, J. A. (1977) Method for detecting carcinogenic organic chemicals using mammalian cells in culture. *Br. J. Cancer*, **36**, 558.

SUGIURA, K., HALTER, C. R., KENSLER, C. J. & RHOADS, C. P. (1945) Observations on rats fed with compounds related to dimethylaminoazobenzene. *Cancer Res.*, **5**, 235.

SUGIMURA, T., SATO, S., NAGAO, M., YAHAGI, T., MATSUSHIMA, T., SEINO, Y., TAKEUCHI, M. & KAWACHI, T. (1976) Overlapping of carcinogens and mutagens. In *Fundamentals in Cancer Prevention*. Ed. Magee, P. B., Tokyo Univ./Park Press, Baltimore.

TERENT'EV, A. P. & PREOBRAZHENSKAYA, M. N. (1959) A method of introduction of substituents into the benzene ring of Indole. II. Preparation of 5-bromo-1-methylindole and 5-amino-1-methyl indole. *Zh. Obshch. Khim.*, **29**, 317. (*Chem. Abstr.*, **53**, 21874e.)

TOPHAM, J. C. & WESTROP, J. W. (1964) Thin-layer chromatography of 4-dimethylaminoazobenzene and some of its metabolites. *J. Chromatog.*, **16**, 233.

U.S. PUBLIC HEALTH SERVICE (1954) Survey of Compounds which have been tested for Carcinogenic Activity. Publication No. 149 and Supplements. U.S. Government Printing Office, Washington DC.

VAN LOON, A., VERKADE, P. E. & WEPSTER, B. M. (1960) Preparation of some 4-dimethylaminoazo derivatives with substituents in the 3- or in the 3- and the 5-position. *Rec. Trav. Chim.*, **79**, 977.

WEISBURGER, J. H., YAMAMOTO, R. S., WILLIAMS, G. M., GRANTHAM, P. H., MATSUSHIMA, T. & WEISBURGER, E. K. (1972) On the sulphate ester of N-hydroxy-N-2-fluorenylacetamide as a key ultimate hepatocarcinogen in the rat. *Cancer Res.*, **32**, 491.

WESTROP, J. W. & TOPHAM, J. C. (1966) The hydroxylation and carcinogenicity *in vivo* of aminoazo dyes. *Biochem. Pharmacol.*, **15**, 1395.

WITT, O. N. & THOMAS, E. G. P. (1883) Researches on the indoline group. *J. Chem. Soc.*, 112.

YAMAMOTO, R. S., GLASS, R. M., FRANKEL, H. H., WEISBURGER, E. K. & WEISBURGER, J. H. (1968) Inhibition of the toxicity and carcinogenicity of N-2-fluorenylacetamide by acetanilide. *Tox. Appl. Pharmacol.*, **13**, 108.

YAMAMOTO, R. S., WILLIAMS, G. M., RICHARDSON, H. L., WEISBURGER, E. K. & WEISBURGER, J. H. (1973) Effect of p-hydroxyacetanilide on liver cancer induction by N-hydroxy-N-2-fluorenylacetamide. *Cancer Res.*, **33**, 454.